# Supplementary material for: High-performance hysteresis-free perovskite transistors through anion engineering
Source: Nat Commun. 2022 Apr 1;13:1741. doi: 10.1038/s41467-022-29434-x (PMC8975846; doi:10.1038/s41467-022-29434-x)
Supplement: Supplementary file 1 — Supplementary information [file 41467_2022_29434_MOESM1_ESM.docx]

*Supplementary information*

**High-performance and hysteresis-free perovskite transistors with anion engineering**

Huihui Zhu^1^, Ao Liu^1^, Kyu In Shim^2^, Haksoon Jung^1^, Taoyu Zou^1^, Youjin Reo^1^, Hyunjun Kim^1^, Jeong Woo Han^2^, Yimu Chen^3^, Hye Yong Chu^4^, Jun Hyung Lim^4^, Hyung-Jun Kim^4^, Sai Bai^5, 6^*, Yong-Young Noh^1^*

^1^Department of Chemical Engineering, Pohang University of Science and Technology, 77 Cheongam-Ro, Nam-Gu, Pohang 37673, Republic of Korea

^2^Department of Chemical Engineering and School of Interdisciplinary Bioscience and Bioengineering, Pohang University of Science and Technology, 77 Cheongam-Ro, Nam-Gu, Pohang 37673, Republic of Korea

^3^Ministry of Industry and Information Technology Key Lab of Micro-Nano Optoelectronic Information System, Harbin Institute of Technology, Shenzhen, 518055, China

^4^R&D Center, Samsung Display Inc., Yongin 17113, Republic of Korea

^5^Institute of Fundamental and Frontier Sciences, University of Electronic Science and Technology of China, Chengdu, 611731, China

^6^Department of Physics, Chemistry and Biology (IFM), Linköping University, Linköping SE-58183, Sweden

*Corresponding to authors: Y. Y. Noh ([yynoh@postech.ac.kr](mailto:yynoh@postech.ac.kr)) and S. B ([sai.bai@liu.se](mailto:sai.bai@liu.se))

**Chemicals.** N,N-dimethylformamide (DMF, anhydrous, 99.8%, Sigma-Aldrich), dimethyl sulfoxide (DMSO, anhydrous, ≥99.9%, Sigma-Aldrich), methylammonium iodide (MAI, ≥99.5%, Xi’an Polymer Light Technology Corp), tin(II) iodide (SnI_2_, anhydrous, beads, 99.99% trace metals basis, Sigma-Aldrich), lead(II) iodide (PbI_2_, 99.9985% metals basis, Alfa Aesar), tin(II) fluoride (SnF_2_, 99%, Sigma-Aldrich), lead(II) bromide (PbBr_2_, 99.99% trace metals basis, Sigma-Aldrich), lead(II) chloride (PbCl_2_, >99.99%, Xi’an Polymer Light Technology Corp), tin(II) chloride (SnCl_2_, 99.99% trace metals basis, Sigma-Aldrich) and chlorobenze (anhydrous, 99.8%, Sigma-Aldrich) were used as received without further purification.

**DFT calculations.** All density functional theory (DFT) calculations were performed using the Vienna *ab Initio* Simulation Package (VASP)^1^. The Perdew-Burke-Ernzerhof (PBE) functional of the generalized gradient approximation (GGA) was used for the exchange-correlation energies^2,3^. Bulk MASnI_3_ tetragonal crystal structure was initially optimized to have the parameters of a = 6.230 Å, b = 6.230 Å, and c = 6.232 Å. Based on this, (5 × 3 × 1) MASnI_3_ slab structures were constructed with 6 layer thickness and ~15 Å vacuum spacing in the direction normal to the surface. Br-doped slab model, MASn(I/Br)_3_, was also created with same parameters. The energy cutoff for the plane-wave basis set was set to be 600 eV, and Monkhorst-Pack 1 × 1 × 1 *k*-point meshes were used^4^. Moreover, the DFT-D3 method was employed to introduce the van der Waals interaction^5^. For the structural optimization, the bottom three layers were fixed, but rest of atoms were relaxed using a conjugate-gradient algorithm until the difference in the total force was < 0.03 eV/Å, and spin-polarization was taken into considerations^6^. VESTA package was used for structural visualization^7^. For the calculation of binding affinity towards I vacancy site, two slab models of pure MASnI_3_ and Br-doped MASn(I/Br)_3_ were employed. For each slab model, I vacancy site was created on the surface, and different halides (X = I^-^, Br^-^, Cl^-^) were placed at the I vacancy site. Affinity towards I vacancy site depending on different halides was calculated using below equations,

$E_{\mathrm{affinity}}=E_{halide passivated}-E_{I_{vac}}-n*\frac{1}{2}E_{halogen\left( g \right)}$,

where $E_{\mathrm{affinity}}$ is the adsorption energy of halide to the I vacancy site on the surface, $E_{halide passivated}$ is the total energy of halide passivated at the I vacancy site on the surface, $n$ is the number of halides being passivated, $E_{anion\left( g \right)}$ is the total energy of halogen gas. For MASn(I/Br)_3_ model, above equation was also applied using total energy of passivated at the I vacancy site of MASn(I/Br)_3_ slab instead of MASnI_3_ slab. In order to simulate the randomness of Br passivation, Br was doped at random locations within the slab layers at Br to I ratio of 0.05.


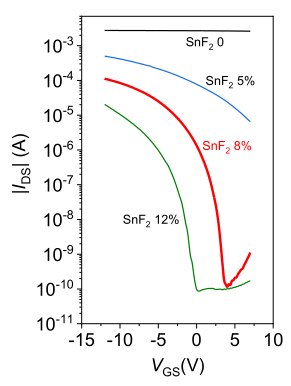


**Supplementary Fig. 1.** Transfer characteristics of pristine MASnI_3_ TFTs *versus* the amount of SnF_2_ additives in the 0.15 M precursor solutions (0~12 mol% with respect to the Sn source) and 8% was the optimized ratio.


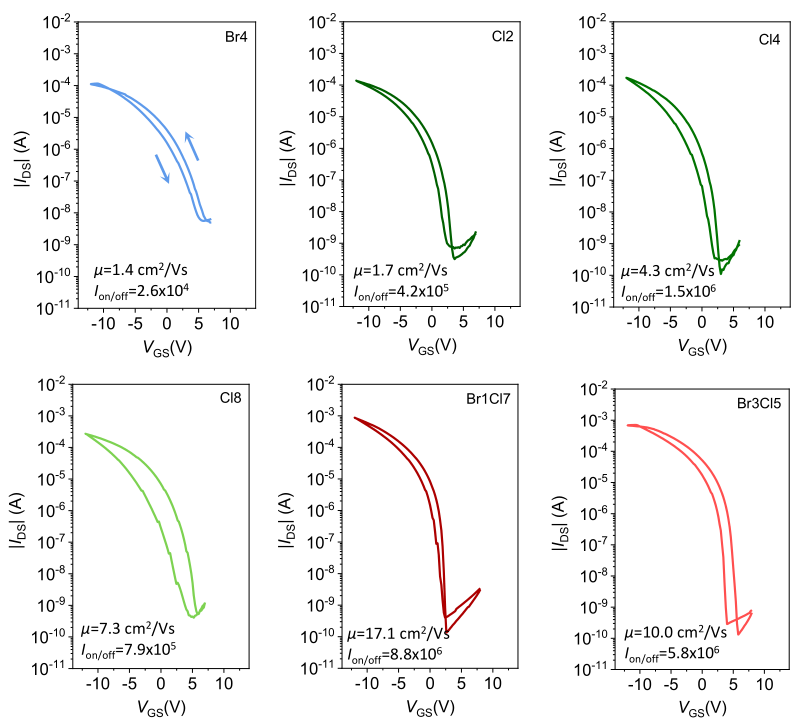


**Supplementary Fig. 2.** The optimizations of halide composition for MASnX_3_ based perovskite TFTs. Br4, Cl2, Cl4, Cl8, Br1Cl7, and Br3Cl5 denote the devices based on channel films processed from precursors of MASn(I_0.96_Br_0.04_)_3_, MASn(I_0.98_Cl_0.02_)_3_, MASn(I_0.96_Cl_0.04_)_3_, MASn(I_0.92_Cl_0.08_)_3_, MASn(I_0.92_Br_0.01_Cl_0.07_)_3_, and MASn(I_0.92_Br_0.03_Cl_0.05_)_3_, respectively.


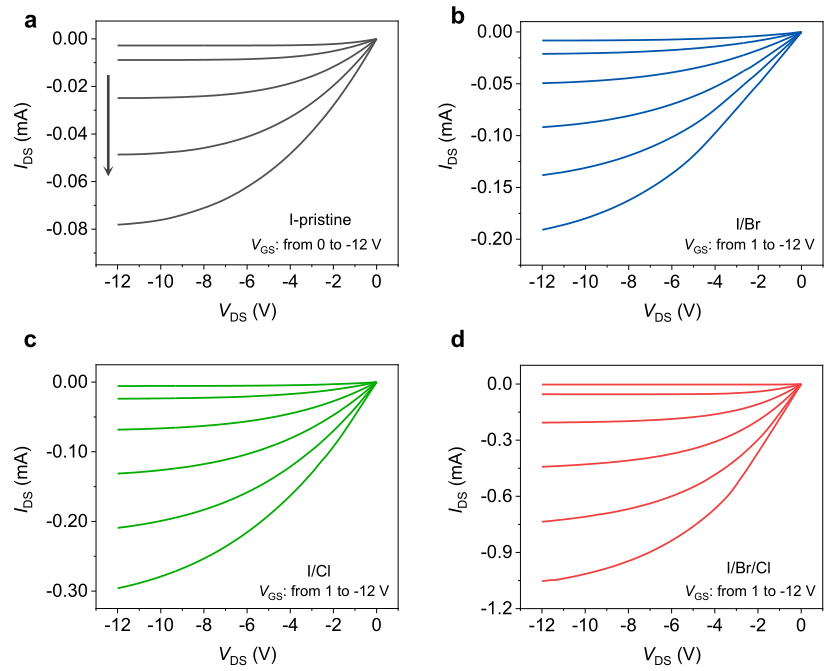


**Supplementary Fig. 3.** Output characteristics of the (a) I-pristine, (b) I/Br, (c) I/Cl and (d) I/Br/Cl perovskite TFTs. I-pristine, I/Br, I/Cl, and I/Br/Cl denote the devices based on channel films processed from precursors of MASnI_3_, MASn(I_0.98_Br_0.02_)_3_, MASn(I_0.94_Cl_0.06_)_3_ and MASn(I_0.92_Br_0.02_Cl_0.06_)_3_, respectively.


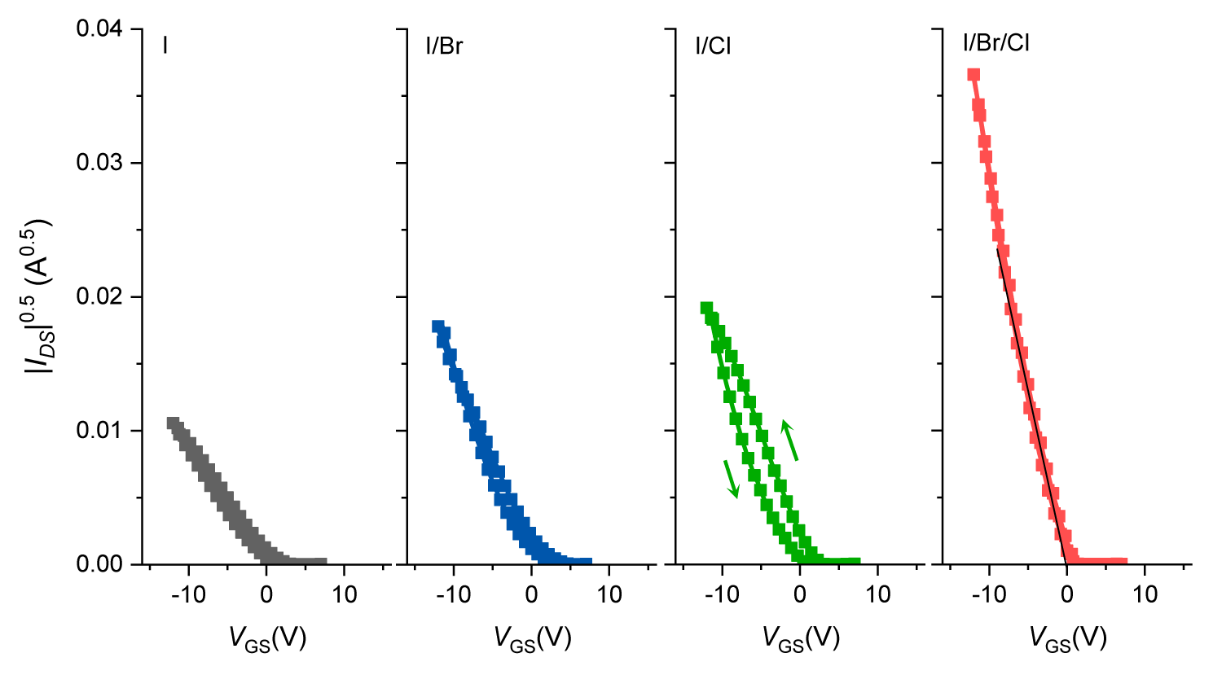


**Supplementary Fig. 4.** |*I*_DS_|^1/2^ of the perovskite TFTs with the I-pristine, I/Br, I/Cl and I/Br/Cl channel films, respectively.


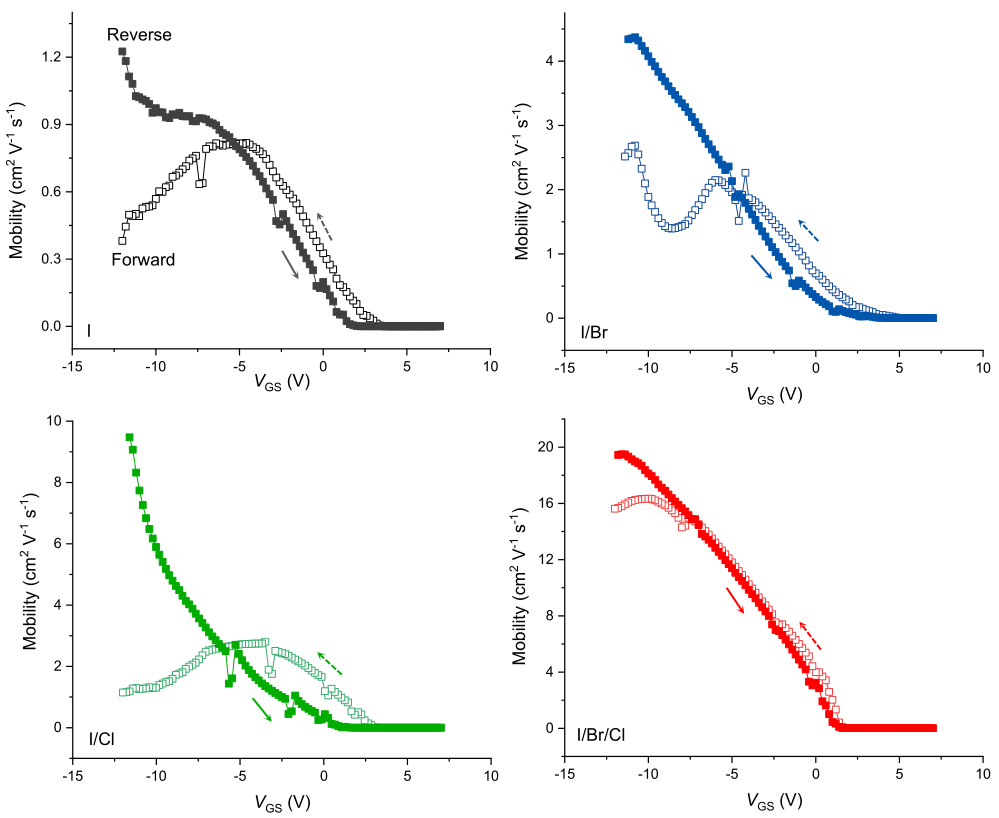


**Supplementary Fig. 5.** Dual-sweep mobilities of the perovskite TFTs with the I-pristine, I/Br, I/Cl and I/Br/Cl channel films, respectively. The maximum values were extraced from reverse and forward sweeps for per device, respectively.


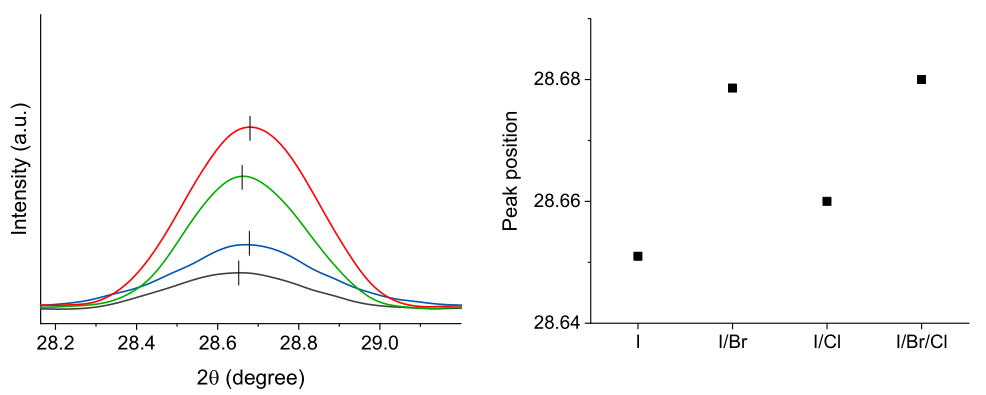


**Supplementary Fig. 6.** XRD peak shift of the perovskite TFTs with the I-pristine, I/Br, I/Cl and I/Br/Cl channel films, respectively.


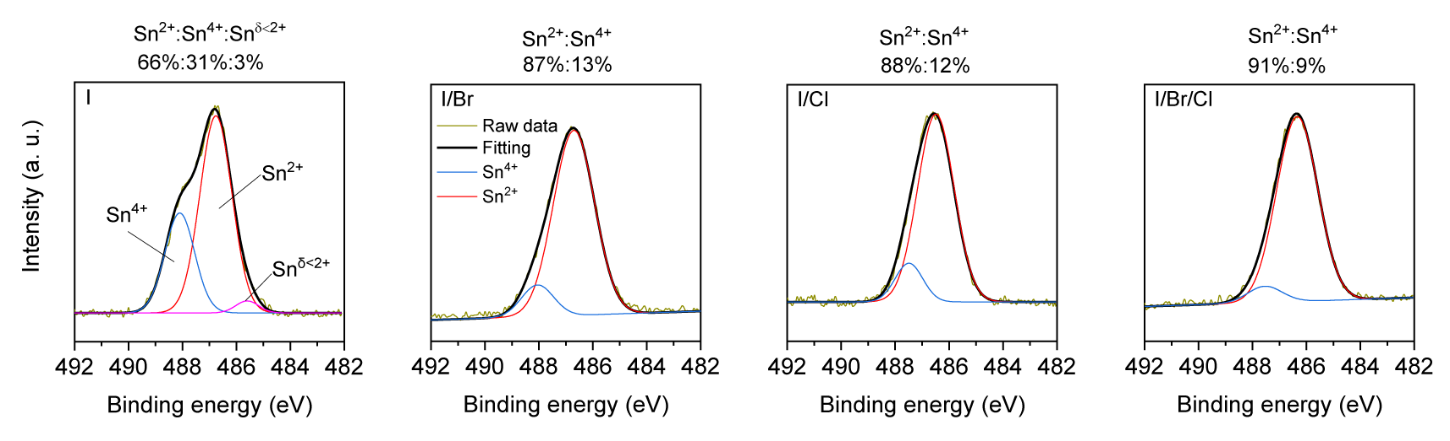


**Supplementary Fig. 7.** Analyses of the Sn 3*d*_5/2_ core level XPS spectra with fitting results for Sn^δ<2+^, Sn^2+^, and Sn^4+^.


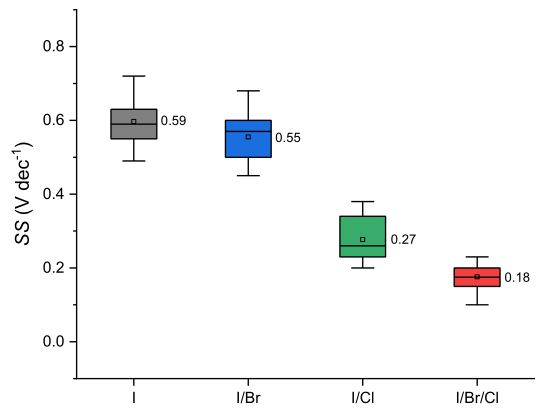


**Supplementary Fig. 8.** Subthreshold swing (SS) statistics from ten individual TFTs with the I, I/Br, I/Cl and I/Br/Cl channel films, respectively. The error bars present standard errors calculated from ten devices per type, and the mean values are labelled.


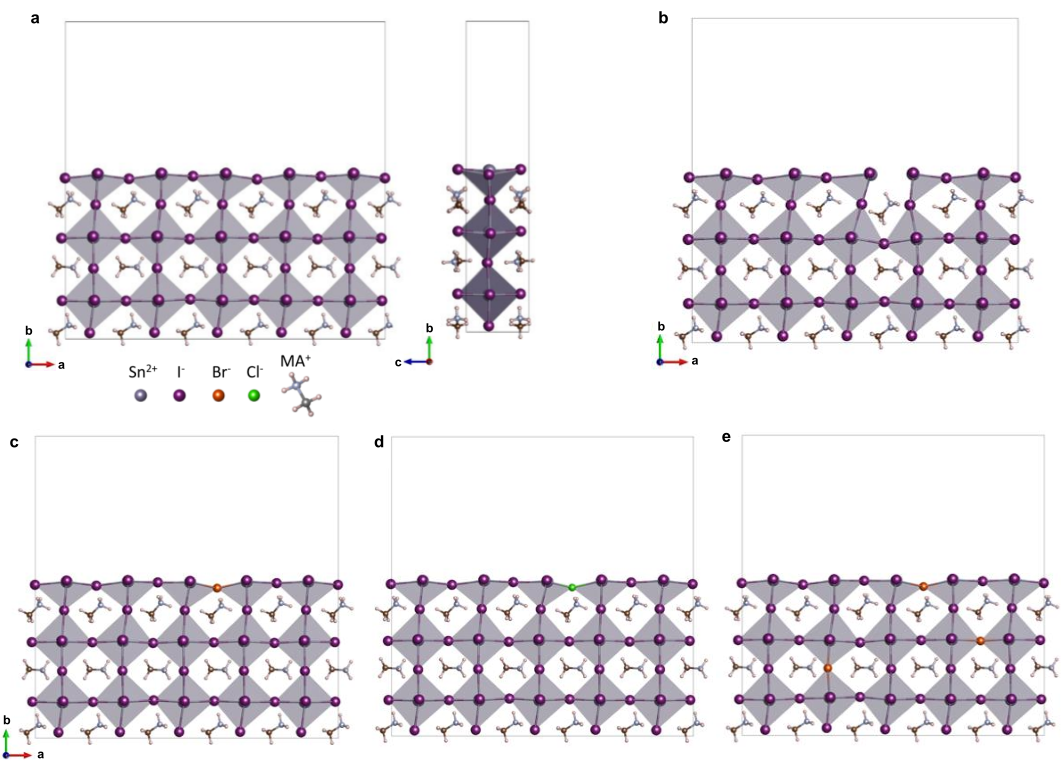


**Supplementary Fig. 9.** DFT slab models of (a) basic MASnI_3_, (b) MASnI_3_ with an iodine vacancy (*V*_I_). Illustrations of *V*_I_ passivation by (c) Br or (d) Cl anions in MASnI_3_, and (e) illustrations of *V*_I_ passivation by a Br anion in MASn(I/Br)_3_ case.


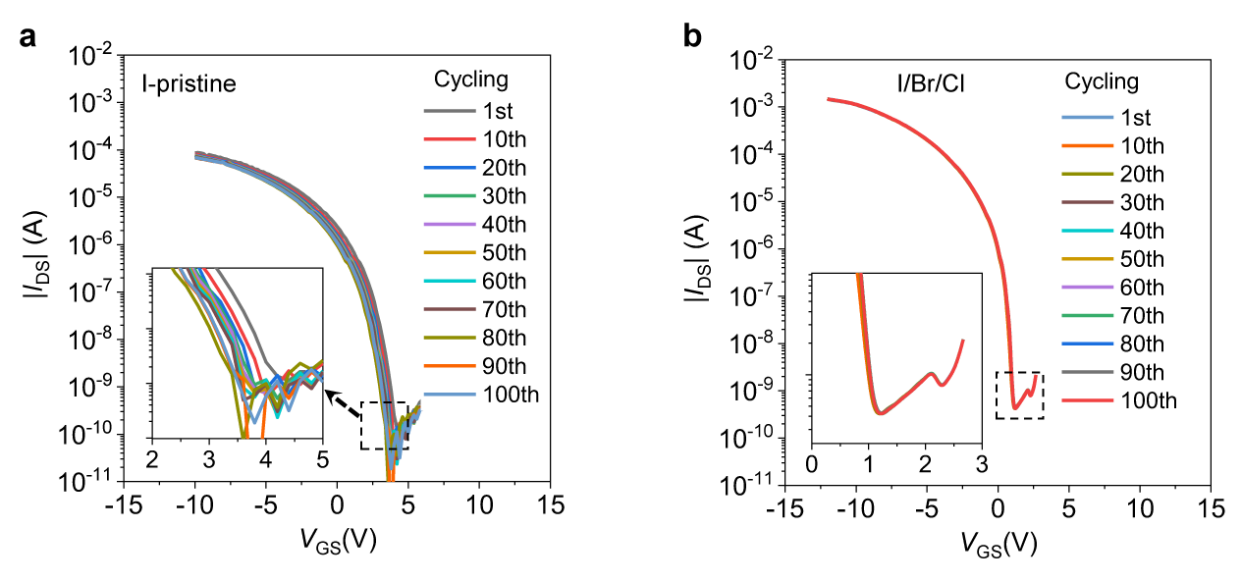


**Supplementary Fig. 10.** Transfer characteristics of (a) I-pristine and (b) I/Br/Cl TFTs under cycling test (*V*_DS_=-12 V).


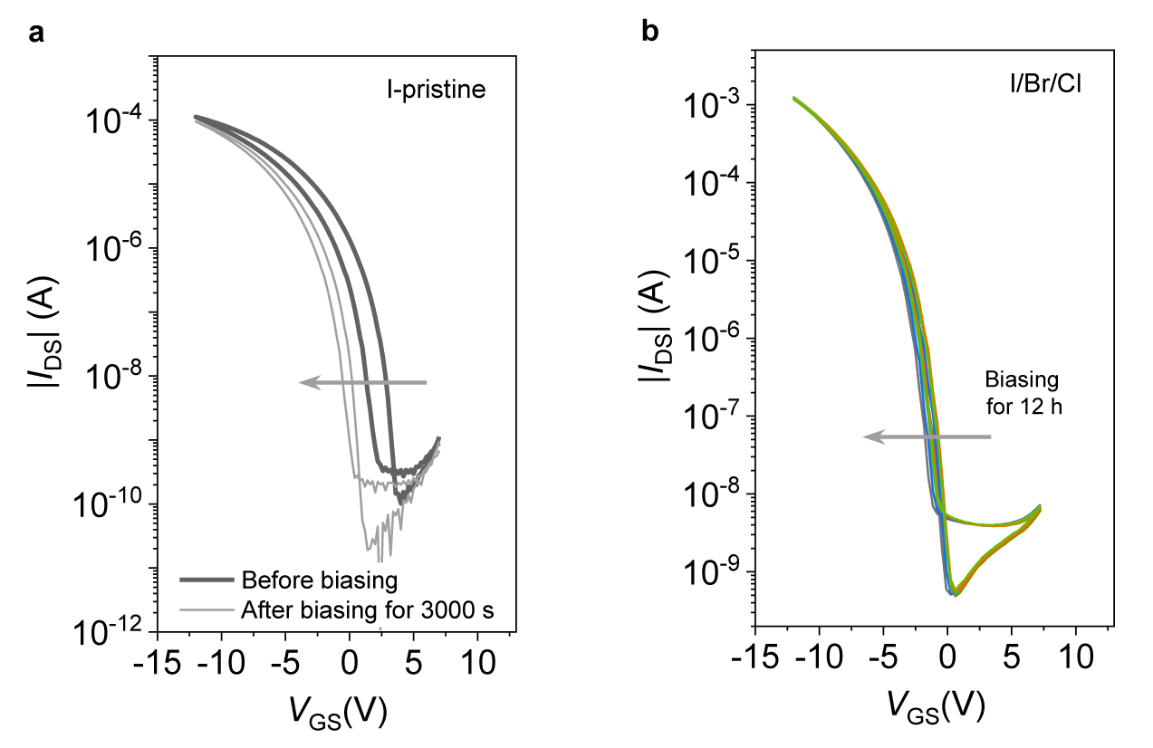


**Supplementary Fig. 11.** Transfer characteristics of (a) I-pristine and (b) I/Br/Cl TFTs under bias (*V*_GS_=*V*_DS_=-12 V).


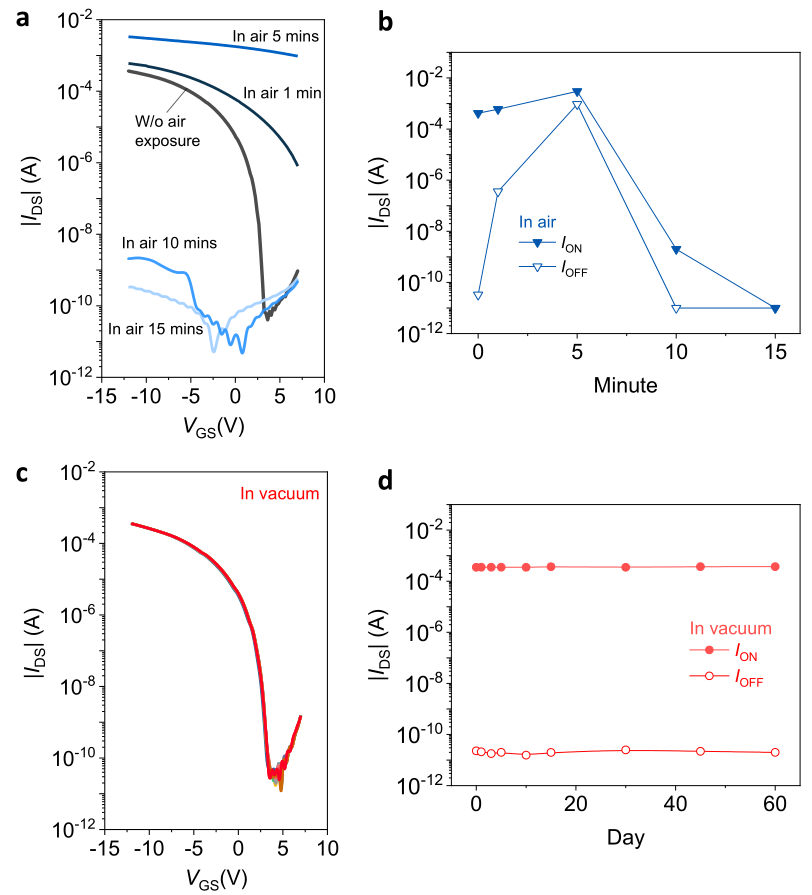


**Supplementary Fig. 12.** (a, b) Air exposure behavior and (c, d) vacuum-stored performance of the perovskite TFTs. (Vacuum condition: ~1×10^-6^ Torr; air condition: ~20 ^o^C, RH ~40%).


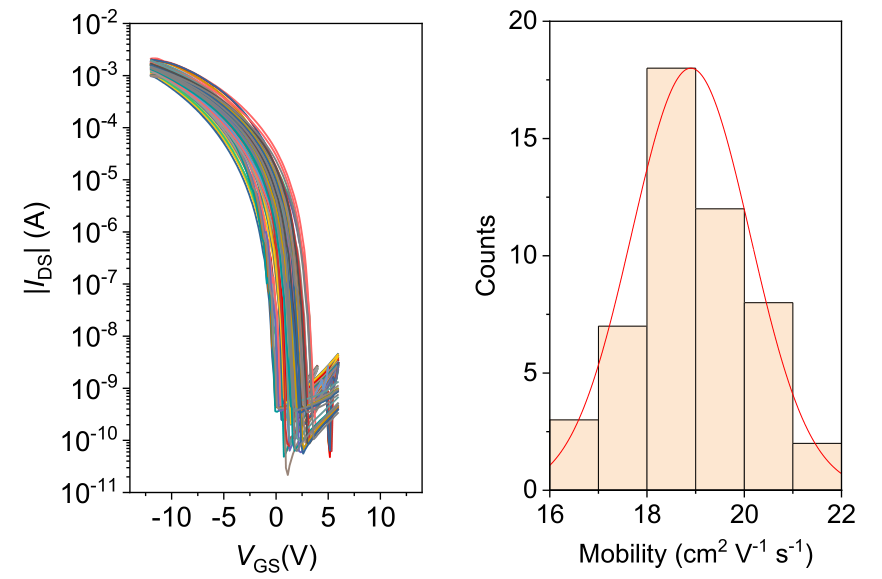


**Supplementary Fig. 13.** Transfer characteristics and mobility statistics of 50 individual I/Br/Cl TFTs. The average mobility extracted from reverse scans is 19±1.2 cm^2^ V^−1^ s^−1^.

**
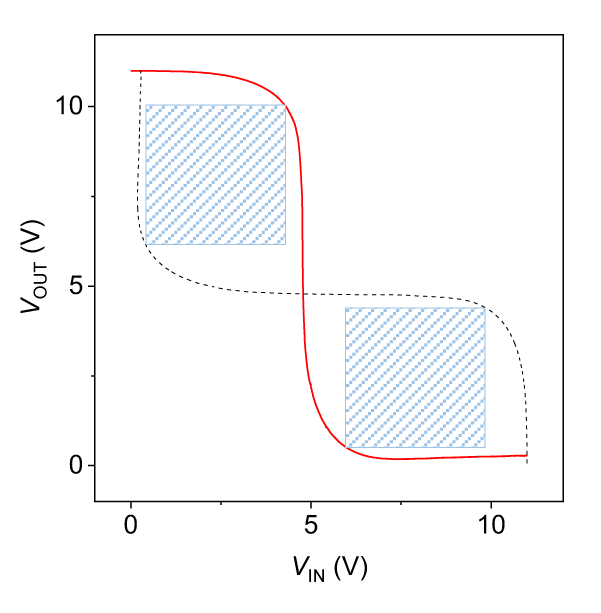
**

**Supplementary Fig. 14.** The butterfly voltage transfer curves plot of the integrated perovskite/IGZO complementary inverter. The black-dash curve was plotted in mirror. The noise margin can be estimated by nesting the largest possible rectangular area inside the butterfly plot, following the maximum equal criterion method^8^.

**Supplementary Table 1.** Performance parameters of Pb-and Sn-based perovskite transistors.

|  | **Perovskite Channel** | **Interlayer/**  **Processing^*^** | **Operation Temp.** | **Mobility (cm^2^ V^−1^ s^−1^)** | | **On/off ratio** | **Year** | | **Ref.** |
| --- | --- | --- | --- | --- | --- | --- | --- | --- | --- |
|  |  |  |  | ***μ*_h_** | ***μ*_e_** |  |  |  |  |
| 3D Pb-based perovskites | MAPbI_3_ | / | 78 K | 2.1 × 10^−2^ | 7.2 × 10^−2^ | ~10^5^ | 2015 | | ^9^ |
|  |  | / | 180 K | / | 1 × 10^−2^ | ~10^4^ | 2015 | | ^10^ |
|  |  | PEIE | 300 K | / | 0.5 | ~10^4^ | 2017 | | ^11^ |
|  |  | Microplate | 200 K | / | 0.3 | ~10^5^ | 2016 | | ^12^ |
|  |  | Microplate, Gra modified | 77 K | / | ~4 | ~10^5^ | 2017 | | ^13^ |
|  |  | Surface cleaning | RT | 1.8 | 3.0 | ~10^5^ | 2020 | | ^14^ |
|  | MAPbI_3−x_Cl_x_ | TTFB-SAM | RT | 1.3 | 1.0 | ~10^3^ | 2015 | | ^15^ |
|  |  | Protein treated | RT | 0.11 | / | ~10^4^ | 2017 | | ^16^ |
|  | Cs_x_(MA_0.17_FA_0.83_)_1−x_Pb(Br_0.17_I_0.83_)_3_ | DTS | RT | 2.1 | 2.5 | ~10^4^ | 2017 | | ^17^ |
|  | RbCsFAMAPbI_3_ | Sol. treated | RT | / | 1.2 | ~10^4^ | 2020 | | ^18^ |
|  | MAPbX_3_,  X = Cl, Br, I | Single crystal | RT | 4.7 | 1.5 | ~10^5^ | 2018 | | ^19^ |
| 2D Sn-based perovskites | (PEA)_2_SnI_4_ | / | RT | 0.6 | / | 10^4^ | 1999 | | ^20^ |
|  | (PEA)_2_SnI_4_ | Melt  processed | RT | 2.6 | / | ~10^6^ | 2002 | | ^21^ |
|  | (PEA)_2_SnI_4_ | MoO_x_, NH_3_I-SAM | RT | 12 | / | ~10^6^ | 2016 | ^22^ | |
|  | (PEA)_2_SnI_4_ | Semi-CNTs hybrid | RT | 1.5 | / | ~10^5^ | 2019 | | ^23^ |
|  | (PEA)_2_SnI_4_ | Precursor- film-FET optimization | RT | 3.5 | / | ~10^6^ | 2020 | | ^24^ |
|  | (PEA)_2_SnI_4_ | Binary sol. | RT | 3.8 | / | ~10^6^ | 2020 | | ^25^ |
|  | (PEA)_2_SnI_4_ | Urea | RT | 4.2 | / | ~10^5^ | 2021 | | ^26^ |
|  | (m-FPEA)_2_SnI_4_  m=2, 3, 4 | / | RT | 0.2~0.6 | / | ~10^5^ | 2001 | | ^27^ |
|  | (4Tm)_2_SnI_4_ | / | RT | 2.32 | / | ~10^6^ | 2019 | | ^28^ |
|  | (TT)_2_SnI_4_ | / | RT | 9.35 | / | ~10^6^ | 2021 | | ^29^ |
| 2D/3D Sn-based perovskite | (FA/PEA)SnI_x_ | / | RT | 0.21 | / | ~10^4^ | 2021 | | ^30^ |
| 3D Sn-based perovskites | MASn(I)_3_ | / | RT | 1.3 | / | 10^6^ | This work | | |
|  | MASn(I/Br/Cl)_3_ | Halide engineering | RT | 19.6 | / | 3×10^7^ |  |  |  |

Note: The perovskite FETs based on mature dielectrics with uncontested capacitance, e.g. SiO_2_, Cytop, PMMA, were summarized here.^31^ ^*^Thin-film channel layers were solution-processed unless otherwise noted. Research on perovskite-based FETs is still at a relatively early stage,^32,33^ and device test and mobility calculation vary in a wide range from lab to lab.^34-38^ It is suggested: i) drain current should be very distinguishable from gate leakage current; ii) capacitance of dielectric layer should be verified; iii) contact resistance between channel layer and source-drain electrodes should be small enough for precise mobility extraction; iv) if dual-sweep hysteresis exists, detailed mobility extraction should be provided; v) device reproducibility should be reasonable.

**References**

1. Kresse, G. & Furthmüller, J. Efficient iterative schemes for ab initio total-energy calculations using a plane-wave basis set. *Phys. Rev. B* **54**, 11169-11186 (1996).

2. Grimme, S. Semiempirical gga-type density functional constructed with a long-range dispersion correction. *J. Comput. Chem.* **27**, 1787-1799 (2006).

3. Sholl, D. & Steckel, J. A. *Density functional theory: A practical introduction*. (John Wiley & Sons, 2011).

4. Monkhorst, H. J. & Pack, J. D. Special points for brillouin-zone integrations. *Phys. Rev. B* **13**, 5188-5192 (1976).

5. Grimme, S., Antony, J., Ehrlich, S. & Krieg, H. A consistent and accurate ab initio parametrization of density functional dispersion correction (DFT-D) for the 94 elements H-Pu. *J. Chem. Phys.* **132**, 154104 (2010).

6. Press, W. H., Flannery, B. P., Teukolsky, S. A., Vetterling, W. T. J. C. & New York, C. U. P., , 839. Numerical recipes: The art of scientific computing((book)). (1986).

7. Momma, K. & Izumi, F. VESTA 3 for three-dimensional visualization of crystal, volumetric and morphology data. *J. Appl. Crystallogr.* **44**, 1272-1276 (2011).

8. Hauser, J. R. Noise margin criteria for digital logic-circuits. *IEEE. Trans. Educ.* **36**, 363-368 (1993).

9. Chin, X. Y., Cortecchia, D., Yin, J., Bruno, A. & Soci, C. Lead iodide perovskite light-emitting field-effect transistor. *Nat. Commun.* **6**, 7383 (2015).

10. Labram, J. G. et al. Temperature-dependent polarization in field-effect transport and photovoltaic measurements of methylammonium lead iodide. *J. Phys. Chem. Lett.* **6**, 3565-3571 (2015).

11. Senanayak, S. P. et al. Understanding charge transport in lead iodide perovskite thin-film field-effect transistors. *Sci. Adv.* **3**, e1601935 (2017).

12. Li, D. et al. Size-dependent phase transition in methylammonium lead iodide perovskite microplate crystals. *Nat. Commun.* **7**, 11330 (2016).

13. Li, D. et al. The effect of thermal annealing on charge transport in organolead halide perovskite microplate field-effect transistors. *Adv. Mater.* **29**, 1601959 (2017).

14. She, X. J. et al. A solvent-based surface cleaning and passivation technique for suppressing ionic defects in high-mobility perovskite field-effect transistors. *Nat. Electron.* **3**, 694-703 (2020).

15. Mei, Y., Zhang, C., Vardeny, Z. & Jurchescu, O. Electrostatic gating of hybrid halide perovskite field-effect transistors: Balanced ambipolar transport at room-temperature. *MRS Commun.* **5**, 297-301 (2015).

16. Ward, J. W. et al. Solution-processed organic and halide perovskite transistors on hydrophobic surfaces. *ACS Appl. Mater. Interfaces* **9**, 18120-18126 (2017).

17. Yusoff, A. R. b. M. et al. Ambipolar triple cation perovskite field effect transistors and inverters. *Adv. Mater.* **29**, 1602940 (2017).

18. Senanayak, S. P. et al. A general approach for hysteresis-free, operationally stable metal halide perovskite field-effect transistors. *Sci. Adv.* **6**, eaaz4948 (2020).

19. Yu, W. et al. Single crystal hybrid perovskite field-effect transistors. *Nat. Commun.* **9**, 5354 (2018).

20. Kagan, C., Mitzi, D. & Dimitrakopoulos, C. Organic-inorganic hybrid materials as semiconducting channels in thin-film field-effect transistors. *Science* **286**, 945-947 (1999).

21. Mitzi, D. B. et al. Hybrid field-effect transistor based on a low-temperature melt-processed channel layer. *Adv. Mater.* **14**, 1772-1776 (2002).

22. Matsushima, T. et al. Solution-processed organic-inorganic perovskite field-effect transistors with high hole mobilities. *Adv. Mater.* **28**, 10275-10281 (2016).

23. Zhu, H. et al. Perovskite and conjugated polymer wrapped semiconducting carbon nanotube hybrid films for high-performance transistors and phototransistors. *ACS Nano* **13**, 3971-3981 (2019).

24. Zhu, H. et al. High-performance and reliable lead-free layered-perovskite transistors. *Adv. Mater.* **32**, 2002717 (2020).

25. Zhu, H. et al. High-performance layered perovskite transistors and phototransistors by binary solvent engineering. *Chem. Mater.* **33**, 1174-1181 (2021).

26. Zhu, H. et al. A lewis base and boundary passivation bifunctional additive for high performance lead-free layered-perovskite transistors and phototransistors. *Mater. Today Energy* **21**, 100722 (2021).

27. Mitzi, D. B., Dimitrakopoulos, C. D. & Kosbar, L. L. Structurally tailored organic-inorganic perovskites:  Optical properties and solution-processed channel materials for thin-film transistors. *Chem. Mater.* **13**, 3728-3740 (2001).

28. Gao, Y. et al. Highly stable lead-free perovskite field-effect transistors incorporating linear π-conjugated organic ligands. *J. Am. Chem. Soc.* **141**, 15577-15585 (2019).

29. Liang, A. et al. Ligand-driven grain engineering of high mobility two-dimensional perovskite thin-film transistors. *J. Am. Chem. Soc.* **143**, 15215-15223 (2021).

30. Shao, S. et al. Field-effect transistors based on formamidinium tin triiodide perovskite. *Adv. Funct. Mater.* **31**, 2008478 (2021).

31. Yang, T. et al. Understanding, optimizing, and utilizing nonideal transistors based on organic or organic hybrid semiconductors. *Adv. Funct. Mater.* **30**, 1903889 (2020).

32. Shining a light on perovskite devices. *Nat. Electron.* **3**, 657-657 (2020).

33. Zhu, H., Liu, A. & Noh, Y.-Y. Perovskite transistors clean up their act. *Nat. Electron.* **3**, 662-663 (2020).

34. Paulus, F., Tyznik, C., Jurchescu, O. D. & Vaynzof, Y. Switched-on: Progress, challenges, and opportunities in metal halide perovskite transistors. *Adv. Funct. Mater.* **31**, 2101029 (2021).

35. Zhu, H. et al. Printable semiconductors for backplane tfts of flexible oled displays. *Adv. Funct. Mater.* **30**, 1904588 (2020).

36. Zhu, H., Liu, A. & Noh, Y.-Y. Recent progress on metal halide perovskite field-effect transistors. *J. Inf. Disp.*, 1-12 (2021).

37. Liu, X., Yu, D., Song, X. & Zeng, H. Metal halide perovskites: Synthesis, ion migration, and application in field-effect transistors. *Small* **14**, 1801460 (2018).

38. Lin, Y.-H., Pattanasattayavong, P. & Anthopoulos, T. D. Metal-halide perovskite transistors for printed electronics: Challenges and opportunities. *Adv. Mater.* **29**, 1702838 (2017).
